# Assessing sleep quality in older adults: A comparison of three measurement approaches

**DOI:** 10.1093/geroni/igaa057.3256

**Published:** 2020-12-16

**Authors:** Amy Berkley, Patricia Carter

**Affiliations:** 1 University of Kansas, Kansas City, Kansas, United States; 2 Capstone College of Nursing, The University of Alabama, Tuscaloosa, Alabama, United States

## Abstract

Discrepancies between subjective and objective sleep measures have been reported for some time; however, it is critical to consider the implications of inaccurate or incomplete sleep assessment for frail older adults who are struggling to maintain independence. To compare sleep assessment methods, we collected objective sleep measurements (via wrist actigraphy), subjective measures via self-report sleep surveys (Pittsburgh Sleep Quality Index; Insomnia Severity Index, Sleep Hygiene Index), and qualitative data through semi-structured audio-recorded interviews, from 8 older adults who self-reported sleep problems while living in a retirement community in southwestern US. Participants’ objective sleep (Total Sleep Time, Sleep Onset Latency, Wake After Sleep Onset, and Sleep Efficiency) and qualitative narratives were congruent, but self-report measures failed to capture several unique sleep problems identified in the sample. Disordered sleep in older adults has been linked to increased incidence of falls, depression and anxiety, cognitive impairment, institutionalization, and mortality, but traditional sleep assessment instruments, designed for the general adult population, fail to capture many of the experiences and causes that are unique to older adults. functioning. A sleep assessment tool designed to measure older people’s sleep experiences could provide more accurate and sensitive data.

